# Mental distress and associated factors among women who experienced gender based violence and attending court in South Ethiopia: a cross-sectional study

**DOI:** 10.1186/s12905-022-01770-6

**Published:** 2022-05-21

**Authors:** Jerusalem Sewalem, Alemayehu Molla

**Affiliations:** Department of Psychiatry, College of Medicine and Health Science, Dilla University, P.O.BOX: 419, Dilla, Ethiopia

**Keywords:** Court, Ethiopia, Gender, Violence, Mental distress

## Abstract

**Background:**

Gender-based violence is an act that has physical, psychological, and sexual consequences for women. It is a widespread issue, particularly in developing countries, and it causes women mental distress. Despite the fact that gender-based violence has a significant impact on mental distress, there have no study in Ethiopia. As a result, the purpose of this study was to determine the prevalence and associated factors of mental distress among mothers who had experienced gender-based violence and were in court.

**Methods:**

A cross-sectional study was conducted on 423 samples. The data was entered into Epi-data version 3.01 and analyzed with SPSS version 21. Binary logistic regression was used, and variables with p-values less than 0.05 were considered statistically significant with regard to mental distress at the respective 95% CI.

**Result:**

The prevalence of mental distress was found to be 59.6% in this study. Mental distress was associated with factors such as a lack of social support, a lack of formal education, a husband's substance use, rural residence, age greater than 33 years, and a low family income.

**Conclusion:**

The prevalence of mental distress is high when compared to the majority of previous findings from other countries. Screening and managing psychological distress in women with a history of violence is preferable, and integrating psychosocial care into court services is recommended.

## Background

Gender based violence is physical, sexual, and psychological abuse of women occurring in public or private life [1]. It is also an act of physical, psychological, and sexual impacts on women by men [2] and is rooted in unequal power relations between women and men, and lead to the mental distress [3]. World wide 35% of women have experienced Gender based Violence (GBV) by someone else [4, 5]. According to WHO report the consequence of GBV was unwanted pregnancies, induced abortions, gynaecological problems, sexually transmitted infections like Human immune virus (HIV), psychological trauma and in the most extreme cases can lead to the death. The problem of GBV and its impact is also high in the developing world [6, 7]. Gender based violence can be explained in four types of abuses including rape, other forms of sexual assault, intimate partner violence (IPV) and stalking [8, 9].

Mental distress is defined as a state of emotional suffering characterized by the undifferentiated combinations of symptoms of depression (e.g., lost interest; sadness; hopelessness) and anxiety (e.g., restlessness; feeling tense) which are sometimes accompanied by somatic symptoms (e.g., insomnia; headaches; lack of energy) [10, 11]. It is also viewed as an emotional disturbance that may have an impact on the social functioning and day-to-day living condition of individuals [12]. The prevalence of mental distress is higher among people detained within the justice system compared to the general population [13–15]. International systematic reviews on the mental health of people with in court show that populations in prisons are multiple times more likely to have several major mental disorders [13, 14]. Mental distress associated with GBV includes mood, anxiety and somatization disorders as well as suicidal behavior [8, 16, 17]. As a severe form of trauma, GBV is an important precipitant of mental disturbance amongst women. There is a gap in knowledge impact of GBV and mental distress and other related mental disturbance in developing countries [17, 18].

Studies done in other countries show the evidence of relationship between GBV and mental distress among women [19, 20]. A study done on lifetime prevalence of GBV and relationship with mental disorders and psychosocial function show that 27.4% women experiencing GBV and for women who experienced three or four types of GBV the rates of mental disorders were 77.3%, including 52.5% anxiety disorders, 47.1% mood disorder, 56.2% substance use disorder and 34.7% suicide attempts [8]. Study done among divorced women showed that about 51.74% of female’s reported decreased standard of life which caused mental distress [21, 22]. Similarly studies showed that lack of emotional support increase the occurrence of mental distress by half and are also precipitating factors for suicide [23, 24]. The study conducted among prisoners showed that psychiatric illness were diagnosed among 63% of inmates [25]. In United States of America 16.3% of jail inmates reported mental problems during their lifetime [26]. There is a bidirectional effect (i.e. that women experiencing abuse are at greater risk of mental distress and that having a mental distress makes one more vulnerable to abuse) particularly for individually within depressive phase [17].

As a result, people with GBV are burdened by social, psychological and economic consequences which lead to poor quality of life [8, 27]. Considering this, it is important to know more about prevalence of mental distress among women who experienced gender based violence which can intern important for wellbeing of mothers. Therefore, this study was conducted to assess prevalence of mental distress among women experienced gender based violence and attending court in southern Ethiopia.

## Methods and materials

### Study design and study period

Institutional based cross-sectional study was conducted from January 1–March 30/2021.

### Study setting

The study was conducted in Gedeo zone which is located 360 km away from Addis Ababa capital city of Ethiopia and 90 km from the regional capital Hawassa. Zone has eight districts and two towns, namely, Dilla and Yirgachefe. Each districts and towns has 1st instance court and one higher level court.

### Population

All adult women exposed to GBV in the past one month or above and attending court were include in the study.

### Sample size determination and sampling technique

The sample size was determined using single population proportion formula at 95% confidence interval and 5% marginal error. We used 50% proportion, since there is no previous study among women attending court after experiencing GVB and adding 10% non-response rate the final sample of the study was 423. Consecutive sampling technique was employed to select study participants during the data collection period.

### Operational definitions

#### Mental distress

According 20-item self-reporting questionnaires (SRQ-20) with yes or no response; if participant response “yes”, we consider they have mental distress and if the response is “no” they have no mental distress for each item. Finally those scoring ≥ 7 will have probable psychological distress [28].

#### Social support

According to Oslo social support scale (Oslo-3) which ranges from 3 to 14, those respondents who score 3–8 were considered as having poor social support, score 9–11 considered as having moderate social support and score 12–14 is considered as having strong social support [29].

#### Current use

Those who use (non-medical use only) substances (alcohol, khat, cigarette, and others) in the last 3 months [30].

#### Ever use

Those who use (non-medical use only) substances (alcohol, khat, cigarette, and others) at least once in their life time [30].

### Data collection method and instrument

The data were collected using structured questionnaires through face-to-face interviews. Mental distress was assessed by using Self-reported questionnaire (SQR-20) which is widely used instrument with the specificity 83% and sensitivity 89.5%, and had good internal reliability (α = 0.78) [28, 31, 32]. Socio-demographic, substance, psychosocial and clinical related factors were also assessed by using semi structured questionnaires. Eight (one for each woreda) BSc degree nurses and four supervisors who can speak both languages (Gedeoffa and Amharic), and well familiar to the local community culture were used as data collectors and supervisors respectively. All women were individually asked to answer the questionnaires in selected interview rooms.

### Data quality control

All above Questionnaires were translated into local language and back translated into English to ensure its consistency. Three day training was given for data collectors and supervisors by the principal investigator on the objective, instrument, consent form, how to maintain confidentiality, data collection procedure, data collection methods, tools, and how to handle ethical issues. Pre-test was conducted among 5% of participants in the same study setting prior to the actual data collection to identify impending problems on data collection tools and the result of the pretest was not included with original data.

### Data processing and analysis

Once all necessary data were obtained, it was checked for completeness and entered using Epi version 3.01 and exported to SPSS version 21 for analysis. The demographic characteristic of participants was computed by using simple descriptive (mean, percentage, frequency, and standard deviation). In addition, binary and multiple logistic regressions were conducted to explore association and to identify independently associated variables. Bivariate and Multivariate binary logistic regression analysis was performed to determine the presence of significant association between explanatory and outcome variables. Finally, variables with P values less than 0.05 were considered statistically significant and strength of the association was presented by adjusted odds ratio with corresponding 95% C.I.

## Results

### Socio demographic characteristics

A total of 423 female participants were included in the study with the response rate of 100%. The mean age (± SD) of the respondents was 32.77 (± 8.068), with age ranging from 18 to 78 years. Among the respondents, majority were in below 32.7 years old. Of the total participants, 290 (68.6%) were protestant religion follower, and 247 (58.4%) were Gedeo in their ethnicity. The majority of the participants were married 408 (96.5%). The educational status of participants indicated that 264 (62.4%) of them attended primary level of education and above. Regarding occupation, 172 (4.7%) participants reported that they are house wife. Large numbers of respondents were from rural 263 (62.2%). The monthly income of respondents was ranging from 50 to 10,000 Ethiopian birr and for majority monthly income was less than 697.8 (70.2%) (Table1).

**Table 1:** Socio demographic characteristics of female experience GBV attending court 2021 (N = 423)

Variable	Frequency (N = 415)	Percent (%)
Age		
Below 32.7(SD = ± 8.068) 32.7 and above	242 181	57.2 42.8
Religion		
Protestant Muslim Orthodox Catholic	290 30 87 16	68.6 7.1 20.6 3.8
Marital status		
Married Single Divorced	408 12 3	96.5 2.8 0.7
Ethnicity		
Gedio Oromo Other(Gurage Sidama, amhara, tigra)	293 27 103	69.3 6.4 24.3
Education status		
Have no formal education Primary Secondary Preparatory College and above	79 118 94 63 61	19 28.4 22.7 15.2 14.7
Occupational status		
Housewife Government employed Private employed Student	172 35 57 159	40.7 8.3 13.5 37.6
Residency		
Urban Rural	160 263	37.8 62.2
Average monthly income		
< 698 ETB ≥ 698ETB	297 126	70.2 29.8

### Clinical and psychosocial factors of the respondents

Regarding the clinical characteristics of the respondents, the majorities were not using contraceptive 221(52.2%). Among participants, 40 (9.5%) of respondents had chronic medical illness and 14 (3.3%) of them had family history of mental illness and 11(2.6%) participants reported as they had family history of suicidal attempt. Regarding social support, 47.5% reported poor social support and 12.5% of participants had post-traumatic stress disorder. Among married women 197(46.6%) reported as their husband had another wife, and 278 (65.7%) reported as their husband use substance (Table 2).

**Table 2:** Clinical and psychosocial factors of the women experienced GBV attend court Dilla Ethiopia, 2021 (N = 423)

Variable	Frequency	Percent (%)
Social support		
Poor Moderate Strong	201 160 62	47.5 37.8 14.7
PTSD		
Yes No	370 53	87.5 12.5
Use contraceptive		
Yes No	202 217	48.2 51.8
Diagnosed medical illness		
Yes No	40 383	9.5 90.5
Family mental illness		
Yes No	14 409	3.3 96.7
Family history suicidal		
Yes No	11 412	2.6 97.4
Husband married other wife (polygamy)		
Yes No	278 113	71.1 28.9

### Life time and current substance use among respondents

Among total participants, 30 (7.1%) of the respondents had history of substance use within their life time. Regarding the current substance use, majority of them reported that they were using alcohol 60 (56.6%), 21(19.8%) of the respondents were smoking cigarette, and 25 (23.6%) were chewing khat with in the past three months (Table 3).

**Table 3:** Substance use characteristics of women experienced GBV attend court in Dilla Ethiopia, 2021 (N = 423)

Variables	Frequency	Percent (%)
Ever alcohol use		
Yes No	30 393	7.1 92.9
Ever cigarette use		
Yes No	6 417	1.4 98.6
Ever khat use		
Yes No	13 410	3.1 96.9
Current substance use		
Yes No	14 409	3.3 96.7
Current alcohol use		
Yes No	6 417	1.4 98.6
Current cigarette use		
Yes No	6 417	1.4 98.6
Current khat use		
Yes No	14 409	3.3 96.7

### Prevalence of mental distress among women experienced GBV and attending court

Over all prevalence of mental distress among women attending court after experiencing gender based violence was 59.6% with 95% CI (55.1–64.2).

### Factors associated with mental distress

After controling potental variables, female with no formal education were 5.44 times more likely to have mental distress as compared with their counter parts [AOR = 5.44, 95% CI (2.47,11.96)]. The odds of having mental distress among women with poor social support was 1.80 times more compared with women with strong social support [AOR = 1.80, 95%CI (1.11, 2.90)].

Mothers whose husband uses substance were about 2.02 times more likely to have mental distress as compared with their opposite groups [AOR = 2.02, 95%CI (1.26, 322)]. Women with age 33 years and above were 1.67 times more likely to have mental distress as compared with women with age less than 33 years ([AOR = 1.67, 95% CI (1.06, 2.62)]. Participants with monthly income of less than 698 were 1.81 times more likely to have mental distress as compared with their counter parts [AOR = 1.81, 95% CI (1.12, 2.93)].

The odds of having mental distress among respondents who live in rural area were 2.32 times higher as compared to those female who live urban [AOR = 2.32, 95%CI (1.43,3.75)] (Table 4).

**Table 4:** Bivariate and multivariate logistic regression analysis of associated factors of mental distress among women experience GBV attend court, 2021

Explanatory variables	Mental distress		COR, (95%CI)	AOR, (95%CI)	P-value
	Yes	No			
Husband use sub					
No Yes	71 181	74 97	1 1.95(1.29, 2.93)	1 2.02(1.26,3.22)	0.003
Social support					
Poor Moderate Strong	103 104 45	97 57 17	1.72(1.12–2.63) 2.49(1.34–4.65) 1	1.80(1.11–2.90) 2.13(1.08–4.22) 1	0.02
Age in year					
33 year 33 and above	158 94	84 87	1 1.74(1.17,2.58)	1 1.67(1.06,2.72)	0.035
Post-traumatic stress disorder					
Yes No	23 229	30 141	2.12(1.18–3.79) 1	0.59(0.31.1.12) 1	
Living place					
Urban Rural	108 144	52 119	1 1.72(1.14,2.59)	1 2.32(1.43,3.75)	≤ 0.001
Occupation					
Housewife Private business Government employ Student	114 35 15 88	58 22 20 71	1.59(1.02,2.47) 1.28(0.69,2.38) 0.61(0.29,1.27) 1	1.02 (0.68, 1.95) 0.98(0.23,1.35) 0.49(0.21,1.14) 1	
Monthly income					
< 698 > = 698	187 65	110 61	1 1.60(1.05,2.43)	1 1.81(1.12, 2.93)	≤ 0.001
Educational level					
Not Join formal education Joined formal education	85 167	74 97	1.50(1.01,2.24) 1	5.44(2.47,11.96) 1	≤ 0.001

## Discussion

Even though there is no evidence in Ethiopia, some studies in developed countries showed that the prevalence of mental distress among women who experienced gender based violence was high. This study was aimed to assess the prevalence of mental distress among women who experienced gender based violence in southern Ethiopia.

The finding of the current study showed that the overall prevalence of mental distress among mothers experienced gender based violence and attending court was 59.6% with 95% CI (55.1–64.2). The finding of the study was similar with studies done in Australia 58% [33]. Hoverer, the finding was higher than studies done in Ruanda in which 19.7%, 10.8% and 8.0% of participants had anxiety, suicide ideation and PTSD respectively [34], Australia 37% [35], United States of America 28.9% [36], and in Spain 32% [37]. Moreover, it was lower than other study done in Jamaica 77.3% [8]. The possible reason for this variation might be due difference study deigns used, timing of study. Sociocultural and sample size variations might be other possible reasons for the discrepancy of prevalence of mental distress among mothers experienced gender based violence and attending court.

Regarding factors associated with mental distress among women who experienced GBV and attending court; husband’s substance use, poor social support, absence of formal education, livening in rural area, age of women 33 years and above and monthly income of less than 698 were significantly associated with mental distress. Studies also showed that GBV is also strongly associated with disability, poor quality of life, unemployment and overall socioeconomic disadvantage among women [33]. The finding was supported with studies done in USA [38] and León [39]. The possible explanation to this might be substance intoxications of husband is risk factors for women to developed metal distress and an environment with good social support from family may have a buffering effect on an individual’s coping mechanism. High levels of social support may protect patients from the negative prognostic consequences of mental distress. Mother’s educational level was associated with psychological distress and this is supported with findings in [40]. The possible reason might be due to that low education lead to difficulty of getting psychosocial resources and more daily hassles, which in turn mediate to mental distress. Rural residence of women was other factors significantly associated with mental distress, but this is not supported with finding conducted in [41].

## Limitation

The limitation of this study might be due to the cross-sectional nature of study design which cannot establish the temporal relationship between outcome variable and its associated factors.

The recalling bias of some factors and inability of including g qualitative component is another limitation of study.

## Conclusion

The prevalence of mental distress among women experienced gender based violence and attending court was high as compared with many of other studies. It is better to screen and manage psychological distress among women with history of violence and integrating psychosocial care in court service is warranted.

## Data Availability

All raw data included in the manuscript can be accessed from the corresponding author through the email address of “JSEWALEM@gmail.com” with reasonable request.

## Abbreviations

AOR: Adjusted odds ratio
CI: Confidence interval
COR: Crude odds ratio
IPV: Intimate partner violence
PHQ-9: Patient health questionnaire
SRQ-20: Self reporting questionnaire
SNNRE: Southern Nation Nationalities and Regions of Ethiopia
